# Mucopolysaccharidosis Type I in Mexico: Case-Based Review

**DOI:** 10.3390/children10040642

**Published:** 2023-03-30

**Authors:** Consuelo Cantú-Reyna, Diana Laura Vazquez-Cantu, Héctor Cruz-Camino, Yuriria Arlette Narváez-Díaz, Óscar Flores-Caloca, Óscar González-Llano, Carolina Araiza-Lozano, René Gómez-Gutiérrez

**Affiliations:** 1 Escuela de Medicina y Ciencias de la Salud, Tecnologico de Monterrey, Monterrey 64710, Mexico; hcruz@genomi-k.com (H.C.-C.);; 2 Medical Department, Genomi-k, Monterrey 64060, Mexico; diannah.cantu@gmail.com (D.L.V.-C.); caraizal@genomi-k.com (C.A.-L.); 3 Centro Médico, ISSSTELEON, Monterrey 64000, Mexico

**Keywords:** Mucopolysaccharidosis I, Mexico, iduronidase

## Abstract

Introduction: Mucopolysaccharidosis type I (MPS I) is a lysosomal storage disease present in 1:100,000 newborns. Variants in the IDUA (alpha-L-iduronidase) gene decrease the enzyme activity for glycosaminoglycans metabolism. MPS I patients exhibit clinical manifestations that fall on the Hurler, Hurler–Scheie, and Scheie syndrome spectrum. Case presentation: We present a male Mexican patient with respiratory exacerbations requiring recurrent hospitalizations. He showed macrocephaly, coarse facies, hepatomegaly, umbilical hernia, and dorsal kyphosis. The sequencing of the IDUA gene revealed the following genotype: c.46_57del12/c.1205G>A. He received combined therapy with hematopoietic stem cell transplantation and enzyme replacement. Mexican case reports were analyzed to estimate the prevalence of the associated genetic variants. Conclusion: Despite the challenges of managing this rare disease in Mexico, our patient benefited from the combined therapy. The discrete clinical manifestations and prompt evaluation by a geneticist were crucial in establishing a diagnosis, enabling an early intervention by a multidisciplinary team. The combination of ERT before and after HSCT provided health benefits to our patient.

## 1. Introduction

Mucopolysaccharidosis type I (MPS I) is an autosomal recessive disorder caused by the deficiency of alpha-L-iduronidase (IDUA, EC 3.2.1.76) [1]. This lysosomal storage disease has an estimated worldwide prevalence of 1 in 100,000 live newborns [2], while an overall prevalence of 0.19 is estimated in Mexico [3].

MPS I is caused by a partial or, more commonly, a total lack of IDUA enzyme activity [2]. The IDUA genetic variations encode a deficient enzyme, which leads to the accumulation of glycosaminoglycans (GAG) in the cells, blood, and connective tissues. The common clinical features of MPS I include corneal clouding, recurrent respiratory infections, cardiomyopathy, valvular compromise, hepatosplenomegaly, joint stiffness, dysostosis multiplex, growth retardation, developmental delay, and intellectual disability [4].

The IDUA pathogenic variants relate to the severity of the disease. Variants causing an undetectable enzyme activity manifest a severe MPS I phenotype (Hurler syndrome, MPS I-H, OMIM 607014). Partial deficiency in the alpha-L-iduronidase enzyme may show an intermediate MPS I (Hurler–Scheie syndrome, MPS I-H/S, OMIM 607015) or an attenuated form (Scheie syndrome, MPS I-S, OMIM 607016).

Worldwide, the severe form of MPS I is more frequent, accounting for ~60% of patients, when compared with the intermediate (23%) or attenuated (13%) forms [5]. In Mexico, it is estimated that 50–80% of the MPS I cases are consistent with the Hurler phenotype [3]. The high prevalence of Hurler syndrome can be explained by the allele frequency (AF) of the nonsense variants c.208C>T (p.Q70X; AF: 0.167) and c.1205G>A (p.W402X; AF: 0.30) [6]. The latter variant even has a frequency of ~50% in Europe and North America, which is in accordance with Celtic migratory movements [5]. Nevertheless, hundreds of variants have been described along the 14 exons of the IDUA gene [1,7].

Patients with the severe form may not show any pathognomonic signs at birth. It is often only in the first year of life that bone abnormalities are clinically observed as coarse facies, macrocephaly, and kyphosis. This may lead to growth arrest in the second year, and, along with further complications, this results in multiorgan dysfunction and death [4].

In addition to the clinical presentation, the elevation of GAGs in urine should be intentionally searched. IDUA enzyme assays and DNA sequencing provide a definitive diagnosis for MPS I.

A diagnosis, based on early symptom recognition, is challenging to achieve since patients are usually limited to the mitigation of clinical manifestations. Currently, newborn screening identifies reduced IDUA enzyme activity at birth, even before any sign associated to the disease [1].

Early therapeutic approaches, such as enzyme replacement therapy (ERT), hematopoietic stem cell transplantation (HSCT), and combined therapy are available for MPS I patients [8]. These therapies have increased the likelihood of patients maintaining their cognitive abilities, reducing clinical manifestations, and experiencing prolonged survival [8].

Several studies have been performed to establish the genotype–phenotype correlation of MPS I [9,10]. Most patients with the severe form are documented with nonsense variants in one or both alleles, while missense or splice-site variants tend to lead to milder forms. However, cases have been reported where patients exhibit a phenotype that differs from what was expected [9]. Hence, it is believed that the phenotype is the result of the presence of genetic and epigenetic factors [9,11]; thus, the same genotype may result in a variable clinical picture.

As a rare disease, MPS I has represented a challenge in Mexico. Treatment is limited, relatively new, and complicated due to the incipient experience in management. Additionally, a need for information regarding genotype–phenotype correlation and prevalence may aid in designing guidelines according to our reality. As part of this effort, our objective in this article is to present the comprehensive treatment of a successful case of a patient diagnosed with MPS I in Mexico.

## 2. Case Presentation

**Figure 1:**
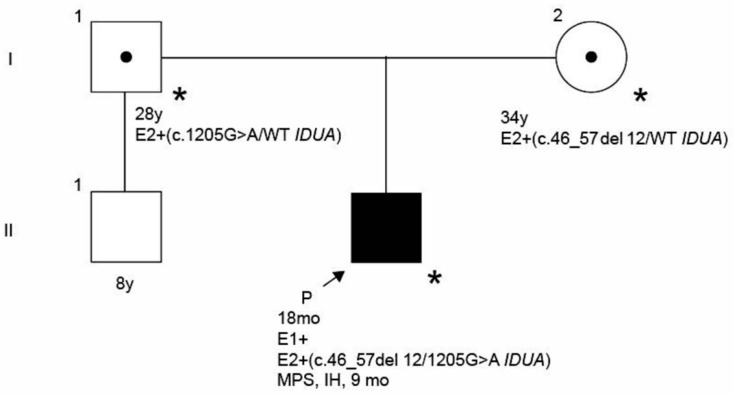
Pedigree of the index patient’s family. Abbreviations: E1 = enzyme assay; E2 = gene sequencing for IDUA; mo: months; MPS-IH: Mucopolysaccharidosis type I, Hurler; WT: wild-type; y: years.

Here, we present a male Mexican patient diagnosed with Hurler syndrome who was asymptomatic at birth. The index patient is the first child of non-consanguineous parents—a 34-year-old woman and the second of a 28-year-old man—(Figure 1), born at 39.4 weeks via an urgent cesarean section after a failed trial of labor. The newborn was macrosomic with a weight at birth of 4040 g (−0.1 months corrected p89) and a length of 54 cm (−0.1 months corrected p96). Primary congenital hypothyroidism screening resulted within the normal limits, but the patient was referred due to an abnormal hearing screening result. The obstetric antecedents comprised a threatened abortion during the first trimester, treated by physical restriction. No family history of a similar disorder, neonatal deaths, or miscarriages was recorded.

The patient presented recurrent respiratory exacerbations requiring multiple hospitalizations. It was at 8 months of life that macrocephaly (p99) with coarse facies, large ear pavilions, short neck, rough breathing, pectum excavatum, hepatomegaly, umbilical hernia, dorsal kyphosis, a lumbosacral Mongolian spot, and bilateral hydrocele were evident in the patient. At that time, the patient presented discreet claw hands but did not present corneal opacity or macroglossia. Despite this clinical picture, the patient’s milestones were achieved in the expected period of time.

The clinical impression led to the request for IDUA activity, which had a significant reduction (0.02 nmol/mL blood/hour via tandem mass spectrometry (MS/MS)) in comparison with the normal reference values (2.02–16.1 nmol/mL blood/hour). Moreover, IDUA gene sequencing was performed, revealing two pathogenic variants, namely c.46_57del12 and c.1205G>A, in heterozygous state. Parental testing for MPS I was recommended to establish the phase of the variants. The mother was identified as a carrier for the c.46_57del12 variant and the father for the c.1205G>A variant. Clinical and molecular evaluations led to the diagnosis of a severe form of MPS I.

**Figure 2:**
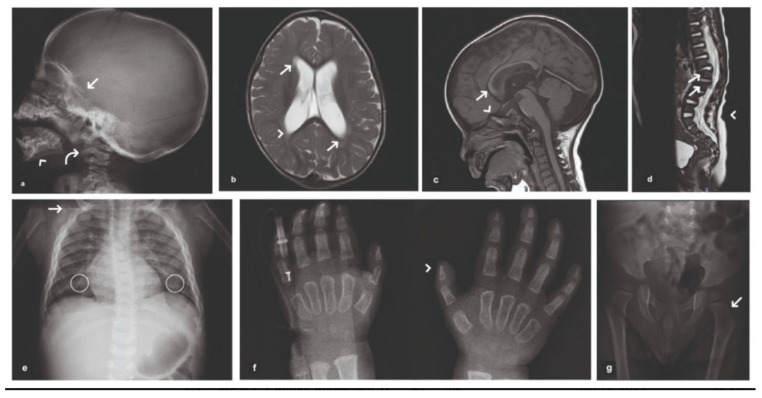
Index patient’s X-ray images. (**a**) Lateral skull X-ray: macrocephaly with abnormal J-shaped sella turcica (arrow); molars unerupted and angulated in both jaws (arrowhead); C2 odontoid process hypoplasia (curved arrow). (**b**) MRI T2: patched hyperintensity areas (arrows); Evans’ index of 0.3; fourth ventricle dilatation (arrowhead). (**c**) MRI T1: thin corpus callosum (arrow); sella turcica dysplasia (arrowhead). (**d**) MRI T2: L1-L2 dysplasia (arrow); exuberant kyphotic curve (arrowhead). (**e**) Anterior posterior chest X-ray: perihilar infiltrate and peribronchial thickening (circles). (**f**) Anterior posterior hand plate X-ray: cone-shaped phalanges (arrowhead). (**g**) Pelvic X-ray: bilateral coxa vara (arrow).

The patient was assessed by a multidisciplinary team, including neurology, ophthalmology, otorhinolaryngology, pneumology, gastroenterology, cardiology, hematology, traumatology, physical rehabilitation, and genetic specialists led by a pediatrician, a nutritionist, and a pediatric therapist. The patient’s imaging findings (Figure 2) showed macrocephaly and a J-shaped sella turcica, pituitary hypoplasia, white-matter hyperintensities, an exuberant kyphotic curve, thoracolumbar scoliosis, bilateral coxa vara, and cone-shaped phalanges. Auditory evoked potential responses revealed a peripheral neuropathy of the median, ulnar, peroneal, and tibial nerves, along with right latency prolongation of III and V waves (70 dB, 114 Hz) and a deficit in conduction replication. Cardiology assessment detected a systolic murmur (II/VI) at the third intercostal left space and a globose left ventricle, which remained under medical surveillance.

Following the Health Institution’s expert panel recommendations, at 10 months old, the patient received the first application of recombinant enzyme L-iduronidase (Aldurazyme^®^; Sanofi S.A., Paris, France) with four intravenous doses (0.58 mg/kg) every 15 days. At 12 months old, he underwent an HSCT. The conditioning regime to prepare the patient for the transplant was as follows: fludarabine at a dose of 30 mg/m^2^ and cyclophosphamide at 350 mg/m^2^, both for 3 days. Additionally, the patient received anti-thymocyte globulin (ATG) at a dose of 2.5 mg/kg for two days and melphalan for a day at a dose of 140 mg/m^2^. The transplant was then performed from the bone marrow of a haploidentical donor (mother) with a cell dose of 4.1 million CD34+ cells/kg of the recipient. On days 3 and 4 post-HSCT, the patient was administered Cyclophosphamide at a dose of 50 mg/kg. Afterward, the same ERT regimen was resumed in the post-transplant period with eight more applications.

**Figure 3:**
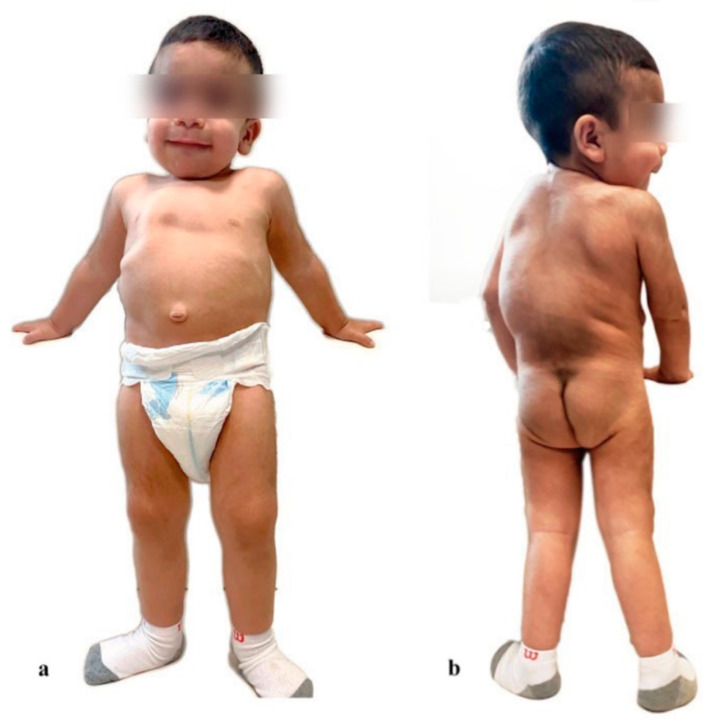
Index patient’s full body anterior and posterior view. (**a**) Anterior view: macrocephaly, pectum excavatum, and umbilical hernia. (**b**) Posterior view: short neck, dorsal kyphosis, and a lumbosacral Mongolian spot.

When the patient was 18 months-old (Figure 3); he completed therapy with immunosuppressants without any adverse reaction. The patient’s weight was 13,400 g (p89), his length was 90 cm (p99), and his head circumference was 49.6 cm (p91). His growth rate, psychomotor development, and length-weight curve remained within normal ranges for his age. Furthermore, his sleeping and eating patterns progressed, his gait and extremity strength was enhanced with physical therapy. His neurodevelopmental milestones were achieved according to his age. The patient’s leukocyte enzyme levels were near the reference range (11.8 hydrolyzed nmol/hour/mg of protein (reference range: 12.0–65.0)), with a full donor chimerism (100%).

At the time of writing, the patient was 4 years-old and had a weight of 17,500 g (p72) and a length of 104 cm (p64). He was generally in good condition and could walk without support. He presented soft skin—not thickened—and the bone manifestations (kyphosis, thoracic deformity and claw hand) have significantly improved. In relation to neurology, the patient presented seizures, which were controlled with carbamazepine. The ophthalmological evaluation showed no sign of eye manifestations related to MPS I. The cardiologist continued recommending surveillance for the innocent heart murmur previously detected. In addition, he attended speech therapy; he knew isolated words, colors, and numbers. He struggled to follow directions, yet was very sociable at school.

His liver, renal, and immunological markers were reported to be within normal limits. In relation to the combined treatment, to this day the chimerism was 100% and enzyme activity was reported within normal limits.

## 3. Discussion

**Figure 4:**
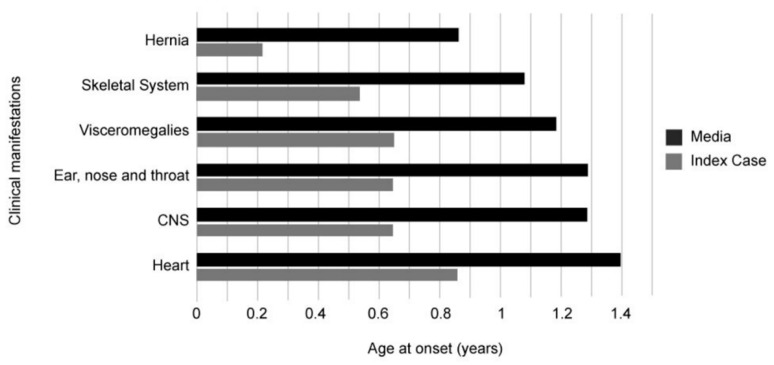
Comparison of symptoms’ median age at onset in patients with Hurler syndrome and the index patient. Abbreviations: CNS, central nervous system.

Here, we present a male patient confirmed with MPS I-H. A multidisciplinary medical approach enabled the rapid identification of radiological signs and symptoms, consistent with other cases presented in the literature (Figure 4) [12,13,14]. Latin American patients with Hurler syndrome have a median age at onset, diagnosis, and first treatment of 1, 1.9, and 5 years, respectively [14]. Conversely, our patient began his treatment at 10 months-old, 4 years earlier than the time reported in the literature.

In relation to the treatment, multiple clinical outcome measurements have elucidated the efficacy of ERT, HSCT, and the combined therapy for MPS I patients [8]. Periodic parenteral ERT is accepted as the gold-standard therapeutic option for this disease [15]. This therapy has demonstrated overall improvements, such as liver size reduction and lower morbidity, and mediates GAG clearance. However, the recombinant human IDUA does not cross the blood–brain barrier; thus, it does not prevent cognitive impairment [5].

On the other hand, HSCT is the ideal treatment approach used to reduce the associated cognitive impairment, as it secretes functional enzymes via the migration of the transplant-derived leukocytes [16]. Three main issues arise from this therapeutic approach. First, several requirements need to be met for a patient to be considered an HSCT candidate (e.g., patients must be less than 2.5 years of age) [7]. Second, there is a high risk of opportunistic infections and mortality due to rejection (graft-versus-host disease). Third, functional enzymes from allogeneic HSCT can be limiting if donors are heterozygous, commonly observed in close relatives [5,16]. Therefore, this treatment is still limited.

ERT before and after HSCT may provide a more favorable environment for donor engraftment, compared to patients receiving the transplant as a first therapeutic approach [15,17]. Along with this, ERT before HSCT is increasingly used as an adjuvant treatment to improve pre-transplant heart and pulmonary conditions [18] and may alleviate symptoms facilitating HSCT candidacy for some patients [7]. This has significant clinical implications since multiple HSCTs are associated with high morbidity and mortality rates [19]. Furthermore, achieving rapid donor chimerism and higher a-L-iduronidase levels through this combination approach could lead to more efficient clearance of GAG from visceral organs, ultimately resulting in improved clinical outcomes for patients with MPS I [15,20].

Evidence suggests that ERT is more effective with a higher number of doses [16,21]. Nevertheless, it is important to consider that access to ERT to treat a patient with MPS I is different in each country. For Mexico, ERT is expensive and the process for obtaining this treatment in the public health sector is generally complex. Therefore, the experience of our patient becomes a starting point for countries facing similar healthcare issues, where an early diagnosis combined with precise, prompt but limited treatment improves the patient’s quality of life.

Molecular findings revealed the pathogenic variants c.46_57del12/c.1205G>A in a compound heterozygous state. This genotype has already been described in the literature with a severe phenotype, consistent with our patient [1]. The allele segregated from the mother was a deletion located at exon 1, translated as p.Ser16_Ala19del (ClinVar ID 92643). This variant has been identified in homozygous and compound heterozygous individuals, both in severe and intermediate MPS I phenotypes [22]. In silico analysis with SIFT predicted a neutral effect with 0.72 confidence. On the other hand, the second allele inherited from the father was located at exon 9, translated as p.Trp402Ter (ClinVar ID 11908). This nonsense variant has been identified in 50% of Caucasian MPS I patients [22]. Similarly in Mexico (mestizo population), the c.1205G>A variant appears to be the most common, accounting for 21% of the alleles included in Table 1, while the c.46_57del12 variant is present in 17%. Furthermore, the lack of consensus regarding the classification of the MPS I subtypes in our country is an obstacle to estimate their specific prevalence.

At present, the patient has had a good quality of life, and will continue to be monitored by a multidisciplinary team; specifically, hematology, neurology, cardiology, gastroenterology, and orthopedics as part of the public health institution service, in line with medical management guidelines [27].

## 4. Conclusions

In this case, the early involvement of the geneticist was crucial in establishing a diagnosis; thus, enabling early intervention through a multidisciplinary team. While this patient had a positive outcome, it is important to note that this level of care is not widely available in Mexico. Patients with MPS I face many challenges in the country, including limited access to healthcare services and treatments, a lack of awareness and understanding of the disease among healthcare professionals, as well as financial barriers.

Our case report provides compelling evidence that an efficient diagnostic process, coupled with timely administration of combined therapy, can significantly improve the prognosis and quality of life for patients with MPS I. Remarkably, our patient achieved favorable outcomes despite receiving a low number of ERT doses, likely due to early diagnosis before the median age. The combination of ERT before and after HSCT provided health benefits to our patient, such as donor engraftment, high chimerism, and bone manifestations improvement, among others. These findings underscore the critical role of early detection and intervention in effectively managing MPS I.

Newborn screening for MPS I has become an important tool for the timely diagnosis of the disease. It enables clinicians to identify infants with MPS I before they start to develop symptoms, allowing for prompt intervention and treatment. While there are challenges to implement newborn screening programs, particularly in resource-limited settings, their benefits in improving patient outcomes cannot be ignored.

Despite the challenges of managing this rare disease, our patient benefited from the combined therapy. Although there is still much to learn about this disease and how best to manage it, our patient’s positive outcome is a hopeful sign and highlights the importance of early diagnosis and access to appropriate treatment.

## Figures and Tables

**Table 1:** IDUA variants and phenotype correlation reported in Mexican patients.

Paatient	Genotype	Protein Variant	Expected Phenotype	Observed Clinical Phenotype	Genotype– Phenotype Correlation
1–3 [22]	c.539G>C/c.1205G>A	p.Trp180Ser/p.Trp402Ter	Various phenotypes [6,22]	Hurler–Scheie	+
4 [22]	c.46_57del12/c.385+1G>C	p.Ser16_Ala19del/intronic variant	Hurler ^b^	Hurler–Scheie	−
5 [22]	c.1598C>G/c.1598C>G	p.Pro533Arg/p.Pro533Arg	Various phenotypes [1,23]	Hurler–Scheie	+
6 [22]	c.385+1G>C/c.1598C>G	Intronic variant/p.Pro533Arg	Hurler ^b^	Hurler	+
7 [22]	c.1205G>A/c.1587_1588insGC	p.Trp402Ter/p.Leu530ArgfsX31	Hurler ^b^	Hurler	+
8 [24]	c.46_57del12/c.46_57del12	p.Ser16_Ala19del/p.Ser16_Ala19del	Hurler–Scheie [23]	Hurler	−
9 [25]	c.965T>A/c.1861C>T	p.Val322Glu/p.Arg621Ter	Hurler ^b^	Detected by NBS	NA
10 [25]	c.701G>C/c.965 T>A	p.Ser234Thr/p.Val322Glu	Hurler–Scheie ^b^	Detected by NBS	NA
11 [26]	c.1898C>G/c.1898 C>G	p.Ser633Trp/p.Ser633Trp	Hurler–Scheie	Hurler	−
Index patient	c.46_57del12/c.1205G>A	p.Ser16_Ala19del/p.Trp402Ter	Hurler [1]	Hurler	+

Mexican patients included in this table were diagnosed with MPS I, for whom their genotype and phenotype were reported. ^b^ The expected phenotype was based on one allele, as it was the only case reported with such genotype. Abbreviations: NA, Not applicable.

## Data Availability

Not applicable.
